# Both serum 25-hydroxyvitamin D and calcium levels may increase the risk of incident prostate cancer in Caribbean men of African ancestry

**DOI:** 10.1002/cam4.457

**Published:** 2015-04-07

**Authors:** Maria D Jackson, Marshall K Tulloch-Reid, Carole M Lindsay, Garrett Smith, Franklyn I Bennett, Norma McFarlane-Anderson, William Aiken, Kathleen C M Coard

**Affiliations:** 1 Department of Community Health & Psychiatry, University of the West IndiesMona, Kingston, Jamaica; 2 Tropical Medicine Research Institute, University of the West IndiesMona, Kingston, Jamaica; 3 Department of Basic Medical Sciences, University of the West IndiesMona Campus, Kingston, Jamaica; 4 Department of Pathology, University of the West IndiesMona Campus, Kingston, Jamaica; 5 Department of Surgery, Radiology, Anaesthesia & Intensive Care, University of the West IndiesMona Campus, Kingston, Jamaica

**Keywords:** 25 hydroxyvitamin D, African ancestry, serum calcium

## Abstract

Circulating 25-hydroxyvitamin D [25(OH)D] concentrations have been associated with both higher and lower risk of prostate cancer (PCa), whereas elevated levels of circulating calcium has been related to higher risks. However, there are few studies that account for effects of both calcium and 25(OH)D concentrations on incident PCa in a black population. We examined these relationships in a case–control study of men 40–80 years old with newly diagnosed, histologically confirmed PCa in Jamaica, a tropical country. Mean serum calcium concentrations was higher among cases (2.32 ± 0.19 mmol/L) than controls, (2.27 ± 0.30 mmol/L) (*P *=* *0.023) however, there were no differences in 25(OH)D by cancer status (cases, 33.67 ± 12.71 ng/mL; controls (32.25 ± 12.59 ng/mL). Serum calcium was not correlated with 25(OH)D (partial correlation: *r,* 0.06; *P* = 0.287). Multivariable-adjusted models showed a positive linear relationship between PCa and serum calcium (OR, 1.12; CI, 1.00–1.25 per 0.1 nmol/L). Serum 25(OH)D concentration also showed a positive association with PCa (OR, 1.23; CI, 1.01–1.49 per 10 ng/mL). The odds of PCa in men with serum 25(OH)D tertile 2 was OR, 2.18; CI, 1.04–4.43 and OR, 2.47 CI, 1.20–4.90 for tertile 3 (*P*_trend_ = 0.013). Dietary intakes of calcium showed no relationship with PCa. Despite the strong relationship between serum calcium and vitamin D the mechanism by which each affects prostate cancer risk in men of African ancestry needs additional investigation.

## Introduction

Cancer of the prostate is the leading cancer site for Jamaican men 1 and few modifiable risk factors have been found to be consistently associated with the disease. Vitamin D has been emerging as one of these modifiable risk factors that play an important role in the development of the prostate cancer (PCa) particularly in men of African origin 2–4; however, the mechanisms for the effect of vitamin D on this disease are not completely understood. In vivo and in vitro studies demonstrate that Vitamin D may work by inhibiting cell proliferation 5 cell migration 6, metastasis and angiogenesis 7,8. However, in Xu et al.'s recent meta-analyses 25-hydroxyvitamin D [25(OH)D] and PCa in predominantly white populations suggests that vitamin D may increase the risk of PCa 4, contradicting the findings from earlier meta-analyses of observational studies which showed no evidence of an association between 25(OH)D and PCa 9–11. Schwartz's systematic review indicated that low 25(OH)D levels increased the risk of fatal PCa 12 and supports ecological studies which suggest higher vitamin D was associated with lower PCa incidence 13. Two nested case–control studies showed that high vitamin D concentrations were associated with increased risk of PCa 2,14 while among African-American men higher vitamin D predicted reduced risk of high-grade disease 14.

Vitamin D is obtained primarily through the synthesis of 7-dehydrocholesterol in the skin exposed to UVB radiation and to a lesser extent, diet (natural foods and fortified products) and supplements 15. Subsequent to synthesis in the skin or ingestion, vitamin D is converted to 25(OH)D in the liver and to the biologically active form, 1,25-dihydroxyvitamin [1,25(OH)_2_D] 16 in the kidneys. 25(OH)D, the predominant circulating metabolite, is influenced by latitude, skin color, and age and is a good marker of vitamin D 17 status. Prostate cells which contain vitamin D receptor (VDRs) can synthesize a small proportion of this vitamin and may respond to 1, 25(OH)_2_D with increased differentiation and apoptosis and decreased proliferation, invasiveness, and metastasis 18.

Vitamin D [1, 25(OH)_2_D], is known to exert control on calcium homeostasis. Elevated levels of calcium could result in increased differentiation and proliferation in PCa cells 19. In experimental studies, calcium has been shown to promote the proliferation and metastasis of PCa cells through the calcium sensing receptors (*CaSR*) on the gland 20,21. Calcium may also work synergistically with hormones such as insulin-like growth factors 22 and estrogens 23 in foods, to influence PCa development. High intake of calcium from dairy products is hypothesized to downregulate the active form of vitamin D, [1,25(OH)_2_D] 24 thereby promoting the development of metastases 7.

There is relative inattention to circulating vitamin D and calcium and PCa risk in populations of African ancestry. Furthermore, the associations of 25(OH)D and calcium concentrations with the incidence of PCa in geographic locations with high irradiance exposure are less clear as few studies have investigated these relationships. In this report we determine the relationship of both circulating concentrations of serum calcium and 25(OH)D with prostate cancer in Jamaica, a predominantly black population (91.6%) 25 and among whom PCa is the leading cancer and the principal cause of cancer mortality 1. We further investigate whether any effect demonstrated is modified by or independent of the other.

## Material and Methods

### Study population

This case–control study has been described in detail in earlier reports 26. Briefly men aged 40–80 years old attending urology clinics at two major hospitals and private urologists' offices in the Kingston Metropolitan area in Jamaica were recruited consecutively and screened for PCa. These sites receive referrals from primary care clinics, hospitals, and private practitioners, island-wide. Routinely, men 40 years and older presenting to the urology clinics/practitioners' offices are asked to do a prostate-specific antigen (PSA) test regardless of presentation.

#### Selection of cases and controls

Cases and controls attended the same urology outpatient clinics/offices and were enrolled consecutively.

##### Cases

Cases were men with newly diagnosed, histologically confirmed PCa and stratified into low-grade and high-grade PCa based on Gleason scores (GS): high-grade, ≥7 and low-grade, GS < 7 27.

##### Controls

Control subjects presented with diagnostic conditions that were primarily related to lower urinary tract symptoms and urinary stones or were referred for investigation and treatment of other suspected genitourinary malignancies or disorders (e.g., male-factor infertility); there were few reports of erectile dysfunction or decreased libido as the primary complaint.

Subjects were assigned as controls based on a digital rectal examination (DRE) and total PSA concentration. Controls were initially defined as men with total PSA<2.0 *μ*g/L (*n* = 176) but subsequently men with total PSA 2.0–4.0 *μ*g/L and free: total PSA>0.15 (*n* = 99) were also included as controls. Data were collected between March 2005 and July 2007.

##### Exclusion criteria

Men with locally advanced and metastatic PCa, previous prostate surgery, with recent severe weight loss, on hormonal treatment or taking Finasteride were excluded from the study.

The study was approved by the Ethics committee of the University of the West Indies and the Ministry of Health. Subjects gave written informed consent prior to enrollment.

### Data collection

Men enrolled in the study were first-time clinic attendees who were interviewed before a pathological diagnosis was obtained. Measurements and interviews were conducted by trained research nurses. Nine hundred and seventy-two men were interviewed and 243 men with newly diagnosed PCa and 275 controls were enrolled; negative biopsy men (*n* = 187) and men who did not meet the inclusion criteria for controls (*n* = 267) were excluded from analyses. In this report, we present information on calcium (224 cases; 248 controls) and 25(OH)D vitamin D (146 cases; 191 controls) for whom a sample of blood was available. Comparison of the demographic characteristics of subjects with and without a sample of blood for calcium investigations showed that they were similar whereas for vitamin D, men for whom data were available were somewhat older (*P *=* *0.062).

### Measurements

The examination included anthropometric assessment (weight, height, waist, and hip circumference) and a questionnaire that included items on medical history and lifestyle variables (cigarette smoking, and physical activity). Medical charts and pathology reports were examined to ensure controls did not have a prior history of cancer. Information on demographic and socioeconomic factors was obtained by questionnaire. Body mass index (BMI) was calculated as weight (kg) divided by height (m)^2^ 28. A nonfasting blood sample was collected for biologic measurements.

#### Laboratory assays

The blood samples collected were placed on ice-packs in a cooler before being processed and stored. Samples from cases and controls were analyzed together within batches to reduce the effect of interassay variability. Laboratory personnel were blinded to disease status.

##### Serum calcium

Serum calcium concentrations were measured on an Abbott C8000 by the Arsenazo 111 method at the University of the West Indies Pathology (Chemical Pathology) Laboratory. The intra-assay coefficient of variation (CV) was 7.9%.

##### Serum 25(OH)D

Serum 25(OH)D concentration was determined by ultra-performance liquid chromatography/tandem mass spectrometry (UPLC/MS/MS) analysis as outlined by 29 using a Waters ACQUITY TQD System (Waters Corporation, Milford, USA) fitted with an ACQUITY UPLC BEH Phenyl column (2.1 × 50 mm × 1.7 *μ*m). The instrument was operated in a positive electrospray ionization mode using MassLynx™ software with data processing by the TargetLynx™ Application manager. Standards were purchased from 1017 West Ninth AvenueKing of Prussia, PA 19406USA (PA). All other reagents and chemicals were obtained from SIGMA-ALDRICH CORPORATION3050 SPRUCE STSAINT LOUIS MO 63103 US.

##### Assay performance

Assay precision was determined by extracting and quantifying five replicates of a quality control sample and the CV was found to be 11%. The recovery of 25(OH)D_3_ was > 90% over the analytical range of the assay. The limit of detection (LOD) for the assay was 0.5 ng/mL and the limit of quantitation (LOQ) was 2.2 ng/mL. The calibration curve was linear over the working range with a correlation coefficient of 0.996.

#### Dietary intakes

Dietary intakes were assessed by use of an interviewer-administered validated 120-item food frequency questionnaire (FFQ) that was developed to assess diet and cancer in the Jamaican population 30. In addition to frequency of consumption for each food item, subjects were asked to estimate the portion size usually consumed by using food models, commonly used household utensils, measuring cups and a measuring tape.

### Statistical analysis

Chi-square statistics or Fisher's exact test were used to examine differences between cases and controls for categorical variables and the Mann–Whitney *U-*test, or *t*-test was used for continuous data. Serum calcium and 25(OH)D were categorized into tertiles based on the distribution among the controls. In addition, 25(OH)D data were analyzed using reference cut-points that included levels of deficiency/sufficiency of <20 ng/mL (deficiency), 20–29 ng/mL (insufficiency) and >30 ng/mL (sufficiency) as suggested by Holick et al. 31, however, we were underpowered (13 cases and 3 controls) to examine relationship between serum calcium above 2.57 mmol/L and 25(OH)D.

Differences between outcome variables (tertiles of serum and dietary calcium and 25(OH)D and socioeconomic variables, medical history, and lifestyle were explored using chi-square statistics and Fisher's exact test for categorical variables and analysis of variance was used for continuous data. Tests for linear trends across tertiles of serum 25(OH)D and calcium were performed using the Wald *χ*^2^ statistic.

Logistic regression was used to estimate odds ratios (OR) and 95% confidence intervals (CIs) for PCa according to per unit change and by tertiles of 25(OH)D and serum and dietary calcium. Analyses were conducted with all PCa cases and compared with all control subjects. In multivariable analysis, adjustment was made for age and BMI (continuous), education (primary or less, secondary, tertiary—a measure of socio-economic status), family history of PCa in first degree relatives (yes, no), physical activity (inactive/moderately inactive, moderately active/active), current smoking (yes, no), and supplement use (primarily vitamins) in our analyses. In addition, we examined whether the association between serum calcium and PCa was modified by 25(OH)D, age, BMI (<25 or ≥25 kg/m^2^), or smoking (current or non-, ex-smoker) by creating a cross-product term between serum calcium and the potential effect modifier. Likelihood ratio tests were used to test for evidence of effect modification. Similar tests were employed for 25(OH)D and included physical activity as a potential effect modifier.

All analyses were performed using the Statistical Package for Social Sciences (SPSS. Statistical Package for Social Scientists, version 19.0. Chicago: SPSS Inc. 2010.) version 19.0. Statistical significance was achieved when *P* < 0.05.

## Results

### Study characteristics

Men with PCa were on average 5 years older than controls (cases, 67.6 ± 7.8; controls, 62.3 ± 10.5 years) (Table 1). With the exception of cases being significantly shorter (cases, 169.6 ± 6.6 cm; controls, 171.5 ± 7.0 cm) and less educated, both groups had similar mean BMI, waist and hip circumference, and waist-hip ratio. Just over one-half of PCa cases were high grade and significantly more men with PCa than controls reported that they were previously screened for the disease.

**Table 1:** Characteristics of cases and controls

	Controls (*n* = 248)	Cases (*n* = 224)	*P*
Socio-demographic			
Age: mean ± SD (years)	62.3 ± 10.5	67.6 ± 7.8	0.0001
Education: % (*n*)			
Primary or less	80.8	90.3	0.003
Secondary or higher	19.2	9.7	
Anthropometry			
Weight (kg)	74.5 ± 14.5	72.4 ± 13.7	0.092
Height (cm)	171.5 ± 7.0	169.6 ± 6.6	0.003
Waist circumference: mean ± SD	84.4 ± 12.7	88.0 ± 12.2	0.590
Body mass index (BMI) kg/m^2^	25.1 ± 4.3	25.1 ± 4.6	0.852
BMI categories			
≤24.99	52.1	54.5	0.576
25.00–29.99	37.1	31.4	
≥30.00	10.8	14.1	
Medical/behavioral			
Cases only: % (*n*)			
Low-grade prostate cancer^1^	–	42.7	
High-grade prostate cancer^2^	–	54.0	
Unknown	–	3.3	
PSA: *μ*g/L median (25th, 75th percentile)	1.6 (0.8, 3.5)	25.8 (12.0, 100.1)	0.0001
Previous PSA screens: %	59.0	88.6	0.0001
Previous DRE: %	75.0	85.9	0.005
Family history of prostate cancer: %	11.1	16.3	0.113
Current smoker: %	16.8	13.2	0.508
Supplement use: %	29.5	23.5	0.185
Serum calcium (nmol/L): mean ± SD (median)	2.27 ± 0.30 (2.31)	2.32 ± 0.19 (2.34)	0.027
Serum 25(OH)D (ng/mL): mean ± SD (median)	32.25 ± 12.59 (30.8)	33.67 ± 12.71 (32.1)	0.300

PSA, prostate-specific antigen; DRE, digital rectal examination.

1 Low-grade cancer indicates Gleason score of <7.

2 High-grade cancer indicates Gleason score of ≥7.

Serum concentrations of calcium were higher among cases (2.32 ± 0.19 nmol/L) than controls (2.27 ± 0.30 nmol/L; *P *=* *0.027). Cases and controls did not differ in mean serum 25(OH) vitamin D level (cases, 33.67 ± 12.59 ng/mL; controls, 32.25 ± 12. 71 ng/mL, *P *=* *0.300) (Table 1).

Table 2 shows tertiles of serum calcium concentration according to selected characteristics of the sample. Mean concentrations of serum calcium in tertiles 1–3 were 2.11 ± 0.13, 2.30 ± 0.04, and 2.48 ± 0.10 mmol/L, respectively. Men in the highest tertile of serum calcium when compared to their counterparts in the lowest tertile reported similar educational achievement and levels of physical activity. Men in tertile 3 were less likely to report a family history of PCa and men in tertile 2 were less likely to report current smoking. Those in the first tertile showed significantly lower mean BMI than men in the upper 2 tertiles.

**Table 2:** Characteristics of men according to tertiles of serum concentration calcium and vitamin D

	Categories of serum concentrations		
Serum calcium (mmol/L)^1^	≤2.23	2.24–2.37	2.38–3.01
Serum calcium (mmol/L) (mean ± SD)***	2.11 ± 0.13	2.30 ± 0.04	2.48 ± 0.10
Participants: *n*	135	172	165
Age: years	64.8 ± 10.1	64.4 ± 9.5	65.4 ± 9.7
Education: %			
Primary or less	83.9	81.5	88.7
Secondary or higher	16.1	18.5	11.4
Family history of prostate cancer: %*	13.5	12.9	10.3
Current smoker: %*	16.5	12.2	15.2
Physical activity: %			
Inactive/moderately inactive	46.7	43.9	44.4
Moderately active/active	53.3	56.1	55.6
Body mass index (BMI) kg/m^2^*	24.1 ± 4.2	25.3 ± 3.8	25.7 ± 4.7
Supplement use: %	23.2	31.7	25.0
Serum vitamin D (ng/mL)^1^	≤27.06	27.07–34.26	34.27–93.20
Serum 25(OH)D, (mean ± SD)***	20.54 ± 5.06	30.62 ± 2.06	45.43 ± 11.31
Participants: *n*	106	117	125
Age: years	64.9 ± 9.7	64.8 ± 10.2	64.1 ± 9.7
Education: %			
Primary or less	77.9	86.1	88.5
Secondary or higher	22.1	13.9	11.5
Family history of prostate cancer: %	14.8	13.9	9.6
Current smoker: %	14.0	16.7	15.2
Physical activity: %			
Inactive/moderately inactive	47.1	41.7	51.6
Moderately active/active	52.9	58.3	48.4
BMI kg/m^2^	25.7 ± 4.9	25.2 ± 4.3	25.2 ± 4.1
Supplement use: %	29.3	26.8	36.6

1 Tertiles based on values of controls.

^*^P < 0.05; ^***^P < 0.0001.

Mean concentrations of serum 25(OH)D in tertiles 1–3 were 20.54 ± 5.06, 30.62 ± 2.06, and 45.43 ± 11.31 ng/mL, respectively (Table 2).The characteristics of men did not vary by other demographic or lifestyle variables. Examination of these variables by 25(OH)D reference cut-points showed that men were similar in characteristics, however, with the small sample size in some groups we may have been underpowered to detect any differences.

### Dietary calcium and PCa

In this report, we excluded from analyses subjects whose reported intakes were outside the range of 800–5000 kcal (*n* = 12) or who provided incomplete information on the FFQ (*n* = 83). A total of 184 cases and 239 controls were used for analyses of diet.

Cases and controls reported similar intakes of calcium (dietary calcium: cases, 1041 ± 411; controls, 1022 ± 412). Table 3 presents information on dietary intakes of calcium stratified by reference cut-points and shows participants reported similar educational achievement and supplement use with increasing dietary calcium intake. However, more men with calcium intakes at the recommended and higher levels reported that they were moderately active/active. Energy, phosphorous, and vitamin D intakes increased with increasing calcium intake.

**Table 3:** Characteristics of men according to the tertiles of dietary intakes of calcium

	Categories of calcium intake (mg/day)		
Dietary calcium (mg/day)^1^	≤800	801–1000	1001–2486
Participants	122	77	224
Dietary calcium (mg/day)***	566 ± 161	900 ± 59	1333 ± 299
Age: years	64.1 ± 9.9	64.3 ± 9.8	64.2 ± 9.9
Education: % (*n*)			
Primary or less	81.4	78.7	86.4
Secondary or higher	18.6	21.3	13.6
Family history of prostate cancer: % (*n*)	10.7	18.4	13.4
Current smoker: % (*n*)	11.5	11.8	19.6
Physical activity: %*			
Inactive/moderately inactive	53.4	51.3	39.6
Moderately active/active	46.6	48.7	60.4
Body mass index (BMI) kg/m^2^	25.5 ± 4.4	25.9 ± 4.7	24.9 ± 4.4
Energy intake (kcal/day)***	2076 ± 710	2749 ± 618	3630 ± 501
Phosphorus intake (mg/day)***	1148 ± 327	1607 ± 306	2165 ± 511
Vitamin D (IU/day)***	284 ± 97	656 ± 114	1256 ± 325

1 Tertiles based on dietary reference intakes (average requirement, 800–1000 mg/day; recommended dietary allowance, 1000–12000 mg/day; upper level of intake, 2000–25000 mg/day). Institute of Medicine FaNB 39.

^*^P < 0.05; ^***^P < 0.0001.

Major food sources of calcium (percentage contribution) to total calcium intake were: cereals (17.0%), vegetables (16.9%), fruits (11.2%), dairy products (9.3%), and seafood (5.8%). Soy or soy products were not significant contributors to calcium intake (0.4%) (data not shown).

Dietary calcium intakes were examined to determine their association with risk of PCa and showed no evidence that dietary intakes were associated with the disease (Table 4). Intakes of ≤1000 mg and >1000 mg calcium daily, showed no relationship with PCa risk (OR, 0.75 CI, 0.44–1.30) (data not shown).

**Table 4:** Odds ratios and 95% CIs for association of dietary calcium intake with total prostate cancer^1^

	Categories of calcium intake (mg/day)			
Dietary calcium (mg/day)	≤800	801–1000	1001–2486	*P*_trend_
Total prostate cancer				
Cases/controls	55/67	36/40	93/132	
Age-adjusted	1.0 (reference)	1.05 (0.58–1.92)	0.83 (0.52–1.32)	0.390
Multivariable-adjusted^1^	1.0 (reference)	1.04 (0.54–1.99)	0.89 (0.46–1.74)	0.717
Multivariable-adjusted^2^	1.0 (reference)	0.95 (0.49–1.85)	0.74 (0.36–1.53)	0.398

1 Adjusted for age, BMI, total energy intake as continuous, BMI, education (primary or less, secondary/tertiary), family history of prostate cancer (no/yes), physical activity (inactive/moderately inactive active, moderately active/active) smoking (nonsmoker, ex-smoker, current smoker) and supplement use (no/yes).

2 Also adjusted for phosphorous and vitamin D intake.

### Serum calcium and 25(OH)D with risk of PCa

Table 5 displays age-adjusted and multivariable-adjusted estimates for serum calcium as a continuous variable with risk of PCa. Serum calcium was positively correlated with PCa diagnosis (multivariable-adjusted: OR, 1.12; CI, 1.00–1.25 per 0.1 nmol/L). There were no significant interactions between PCa, calcium and any of the other factors examined including age, BMI, or a family history of PCa.

**Table 5:** Odds ratios and 95% CIs for association of serum calcium^1^ and 25 (OH) vitamin D^1^with total prostate cancer

	Categories of serum concentrations			*P*_trend_	Serum calcium per 0.1 nmol/L
Serum calcium (mmol/L)^1^	≤2.23	2.24–2.37	2.38–3.01		
Total prostate cancer					
Cases/controls	61/78	82/90	82/83		225/251
Age-adjusted OR (95% CI)	1.0 (ref.)	1.16 (0.72–1.86)	1.24 (0.77–2.00)	0.388	1.09 (0.97–1.23)
Multivariable-adjusted^2^^,^^3^	1.0 (ref.)	1.26 (0.75–2.12)	1.35 (0.80–2.29)	0.269	1.12 (1.00–1.25)
					25(OH)D per 10 ng/mL
25 (OH) vitamin D (ng/mL)^1^					
Total prostate cancer	<27.06	27.07–34.26	34.27–93.20		
Cases/controls	41/65	52/65	60/65		153/195
Age-adjusted OR (95% CI)	1.0 (ref.)	1.29 (0.73–2.27)	1.68 (0.96–2.94)	0.068	1.17 (0.98–1.40)
Multivariable-adjusted^2^^,^^3^	1.0 (ref.)	2.18 (1.04–4.43)	2.47 (1.20–4.90)	0.013	1.23 (1.01–1.49)

1 Tertiles based on values of controls.

2 Adjusted for age and BMI as continuous, education (primary or less, secondary, higher), family history of prostate cancer (no/yes), physical activity (inactive/moderately inactive active, moderately active/active), smoking (nonsmoker, ex-smoker, current smoker) and supplement use (no/yes).

3 Also adjusted for 25(OH)D and serum calcium as appropriate.

With control for age, education, smoking, BMI, supplement use, and family history of the disease, men in the second and third tertiles of serum 25(OH)D had an increased odds of PCa (tertile 2: OR, 2.18; CI, 1.04–4.43) (tertile 3: OR, 2.47 CI, 1.20–4.90; *P*_trend_ = 0.013) (Table 5). As a continuous variable, higher 25(OH)D was related to increased likelihood of PCa (OR, 1.23; CI, 1.01–1.49 per 10 ng/mL). We investigated the relationship between 25(OH)D and PCa using cut-points based on reference values (<20, 20–29, and >30 ng/mL; cases/controls: 16/27, 44/67, 93/103, respectively). Examination of 25(OH)D in which men in lowest tertile of the metabolite served as the reference group showed that men in the higher tertiles had nonsignificant increased odds for PCa (tertile 2: OR, 1.42; CI, 0.52–7.94) (tertile 3: OR, 2.47; CI, 0.97–6.28). Partial correlations (age-adjusted) showed no linear relationship between serum concentrations of vitamin D and calcium (*r*, 0.09; *P* = 0.287).

## Discussion

In this study of men of predominantly African ancestry, we investigated the independent associations of serum calcium and 25(OH)D on PCa risk. We found a dose-dependent relationship with serum 25(OH)D and PCa. Our data showed that each 10 ng/mL increase in 25(OH)D was associated with a 23% increased risk PCa. Serum calcium was associated with a 12% increased risk of PCa for 0.1 mmol/L difference in the metabolite. Serum calcium showed no linear relationship with 25(OH)D (*r*, 0.09; *P* = 0.287) and there was no evidence of interaction between vitamin D and serum calcium and the risk of PCa. Thus, when considered independently, serum calcium and 25(OH)D showed linear associations with PCa risk and appeared to function independently of the other.

Concentrations of serum calcium found in this study compared well to men in the National Health and Nutrition Examination Survey (NHANES) 31,32 and the Malmo Preventive Project 34. Our findings that higher serum calcium concentrations increased the risk of incident PCa are consistent with laboratory studies that show that calcium promotes growth of PCa cells 21. There are few epidemiologic studies on serum calcium and incident PCa 32,34–37. The Malmo Diet and Cancer Study cohort showed a positive relationship between albumin-adjusted calcium and PCa among men aged 55–65 with a BMI <25.0 kg/m^2^ (relative risk, 2.07; CI, 1.08–3.97) 35. Halthur et al., found that, whereas there was no evidence of an overall association between prediagnostic concentrations of serum calcium and risk of PCa in Swedish men (HR, 0.94; 95% CI, 0.81–1.09), young (<45 years) overweight men appeared to have decreased risk of developing PCa 34. Using the prospective cohorts of the first NHANES and the NHANES Epidemiologic Follow-up Study in which 85 cases were identified, Skinner and Schwartz 32 reported that serum calcium was not a significant predictor of incident PCa (HR, 1.31; 95% CI, 0.77–2.20). In contrast, data from the Swedish Apolipoprotein MOrtality RISk study suggested a protective association with calcium or albumin-corrected calcium and PCa risk (HR, 0.91; 95% CI, 0.85–0.98) 37. Bristow et al.'s meta-analysis of randomized controlled trials of calcium supplements on cancer risk showed that supplementation was not associated with risk of overall cancer risk, but for the few events of PCa they recorded an inverse relationship with calcium supplementation (RR, 0.54; 95% CI, 0.30–0.96) 38.

We found nonsignificant relationships between dietary calcium intake and PCa whether using absolute cut-points or by calcium intake tertiles. The mean dietary calcium intake (1000 mg/day) of participants satisfied the Institute of Medicine Recommended Dietary Allowance for men aged 51–70 years 39 with 2.7% of subjects reporting extremely high intakes, that is, ≥2000 mg/day. Huncharek et al.'s meta-analysis of cohort studies showed a weak positive relationship between dietary calcium and PCa risk and found no association in case–control studies (RR, 1.04; 95% CI, 0.90–1.15) 40. More recently, Williams et al.'s study of US veterans and healthy controls showed that total calcium intake (median intake, 708 mg/day) was protective of PCa among Blacks but not Whites 41. Rowland et al.'s study of African Americans and Whites showed that low calcium intake (<604 mg/day) was inversely related to PCa risk among men with the VDR Cdx2 GG genotype that has been linked to low calcium absorption 42.It is possible therefore, that the increased risk observed in this study may be related to factors such as the genetic variation in *CaSR* genes that control serum calcium levels 43.

Our finding of vitamin D as a risk factor for PCa is consistent with Xu's recent meta-analysis that showed higher 25(OH)D concentration increased the risk of PCa (OR, 1.17; 95% CI: 1.05–1.30; *P *=* *0.004) 4. Earlier meta-analyses by Gandini 9, Gilbert et al. 10 and Yin et al. 11 highlighted nonsignificant relationships. In the Selenium and Vitamin E Cancer Prevention Trial (SELECT) the association with plasma vitamin D was U shaped indicating that both high and low vitamin D concentrations were associated with PCa and among African-American men high concentration of the metabolite predicted reduced risk of high-grade disease 2. Weinstein et al.'s examination of the association between 25(OH)D and the primary transporter of vitamin D compounds in the circulation, vitamin D-binding protein (DBP) and PCa showed that serum vitamin D was associated with higher risk of PCa (OR, 1.47; 95% CI: 1.07–2.02), and the association was stronger when DBP levels were higher (OR, 1.81, 95% CI: 1.18–2.79) compared with lower (OR, 1.22; 95% CI: 0.81–1.84; *P*_interaction_ = 0.04) thereby indicating that DBP modified the association between 25(OH)D and PCa 44.

In the present study, 2.9% of subjects (cases, 4; controls, 6) had 25(OH)D concentrations ≤12 ng/mL; 12.4% and of men were 25(OH)D < 20 ng/mL) indicating levels of deficiency (data not shown). At higher concentrations ∽8% of subjects (cases, 12; controls, 16) had 25(OH)D > 50 ng/mL hence the group may be underpowered to detect an association using these categorizations. Murphy et al.'s examination of prostate biopsy outcomes with 25(OH)D reported that 25(OH)D < 20 ng/mL was associated with increased odds of PCa diagnosis on biopsy 45. Our findings confirm reports that suggest that 25(OH)D increases the likelihood of PCa. It is unclear, however, the extent to which circulating 25(OH)D reflects intraprostatic vitamin D concentrations 12.

Serum vitamin D concentrations in our population were comparably higher than levels reported in Caucasians in Europe 46–48 and men of African ancestry in the United States 46–49 but compared well to men in similar geographic location 50. In this study, almost 60% of men had 25(OH)D concentrations at biologically normal levels (≥30 ng/mL); 0.3% of subjects had 25(OH)D concentrations > 90 ng/mL. Observational studies suggest that pigmentary characteristics such as dark skin are likely to inhibit vitamin D synthesis as melanin blocks the initial conversion of 7-dehydrocholesterol to cholecalciferol, the precursor of 25(OH)D in the skin 51. Genetic studies of ancestry and 25(OH)D insufficiency show an inverse relationship between African ancestry and 25(OH)D 49. Gilbert et al.'s study in the UK reported that with adjustment for potential confounding factors such as age and skin pigmentation, men with exposures suggesting reduced levels of cutaneous vitamin D synthesis had lower risk of advanced or high-grade disease and further suggested that men with olive/brown skin were at increased risk of PCa 52. Jamaica is a tropical environment with high solar exposure throughout the year. With geographic coordinates of 18°15′N and 77°30′W, the island records on average ∽8.2 h of sunlight daily hence seasonal variability is not expected to influence the availability of 25(OH)D. Gilbert et al.'s meta-analysis of life course exposure to sunlight with PCa showed weak evidence that low exposure to sunlight was related to PCa risk 52. At present, a role for 25(OH)D in modifying PCa has not been well defined and it is unclear whether high 25(OH)D status is causally related to the disease or a marker of ill-health. There is need to identify a clear biological relationship between high vitamin D and PCa risk.

### Strengths/limitations

We examined the association of serum calcium and 25(OH)D with overall PCa in a predominantly Afro-ethnic population with high UV exposure and is one of the first studies to examine these relationships in the Caribbean. We measured serum 25(OH)D, an indicator of dietary intake and sun exposure. Our data reflected the consumption of multivitamins in general and did not ask specific questions on vitamin D supplements, which could affect the values. We did not evaluate for the presence of renal or liver diseases or hyperparathyroidism in our subjects which may also affect serum Vitamin D and calcium. These conditions are associated with low vitamin D levels, 53–55 however, we do not believe that it resulted in a detection bias as 12.4% of men had 25-(OH)D concentrations <20 ng/mL.

Similar to other epidemiologic studies, there is uncertainty of the extent to which 25(OH)D reflects intraprostatic vitamin D levels as 1,25(OH)_2_D through it local production in prostate cells expressing 25(OH)-1*α*-hydroxylase enzyme 56. Additionally, it is possible that 25(OH)D may be a marker for other factors such as insulin-like growth factor-1 which associated with 25(OH)D and has been related to PCa 57. We measured a single sample of 25(OH)D and calcium and samples representing different time points are likely to give more precise estimates of exposures. Our study was case–control in design hence a temporal relationship between dietary intakes, serum calcium, and vitamin D and PCa cannot be established, however, the design of the study with the use of incident PCa and assessment of the participants prior to any knowledge of a PCa diagnosis strengthen our conclusions. Our findings are valid within an African ancestry population and cannot be extrapolated to other ethnic groups, given the previously reported heterogeneity by pigmentary characteristics 17.

## Conclusion

Our results suggest that serum calcium and 25(OH)D may independently increase the likelihood of PCa among men of African ancestry. Well-designed studies (specifically cohort studies) among Blacks are required to confirm the relationship between circulating 25(OH)D and calcium in the development of PCa in Blacks and explore possible mechanisms in the causal pathway.
